# KCl ultra-thin films with polar and non-polar surfaces grown on Si(111)7 × 7

**DOI:** 10.1038/srep08223

**Published:** 2015-02-04

**Authors:** Igor Beinik, Clemens Barth, Margrit Hanbücken, Laurence Masson

**Affiliations:** 1 Aix Marseille Université, CNRS, CINaM UMR 7325, 13288 Marseille, France

## Abstract

The growth of ultra-thin KCl films on the Si(111)7 × 7 reconstructed surface has been investigated as a function of KCl coverage and substrate temperature. The structure and morphology of the films were characterized by means of scanning tunneling microscopy (STM) under ultra-high vacuum (UHV) conditions. Detailed analysis of the atomically resolved STM images of islands grown at room and high temperatures (400 K–430 K) revealed the presence of KCl(001) and KCl(111) islands with the ratio between both structures depending on the growth temperature. At room temperature, the growth of the first layer, which covers the initial Si(111)7 × 7 surface, contains double/triple atomic layers of KCl(001) with a small fraction of KCl(111) islands. The high temperature growth promotes the appearance of large KCl(111) areas, which are built up by three atomic layers. At room and high temperatures, flat and atomically well-defined ultra-thin KCl films can be grown on the Si(111)7 × 7 substrate. The formation of the above mentioned (111) polar films is interpreted as a result of the thermally activated dissociative adsorption of KCl molecules on Si(111)7 × 7, which produces an excess of potassium on the Si surface.

The growth of thin alkali halide films on metal^1^,^2^,^3^,^4^,^5^,^6^,^7^ and, in a lesser extent, on semiconductor substrates^8^,^9^,^10^ has been the subject of many studies for the last two decades. Apart from a fundamental interest in such films, an emerging topic concerns the formation of supramolecular^11^,^12^,^14^ and covalent^15^ assemblies on such substrates for rising applications in organic electronics or molecule-based sensing devices since it has been recently shown that ultrathin NaCl layers grown on metal surfaces can be beneficially used for the efficient electronic decoupling of adsorbates from the conductive substrates^16^,^17^,^18^.

For this purpose, the formation of atomically well-defined flat layers with a low density of defects is of prime importance. In most of the cases, the initial growth stage of alkali-halide thin films begins with the nucleation of (001) islands with a non-polar surface termination (see Refs. 12 and 13 for a description of polar and non-polar surfaces of alkali halide crystals). In this respect it is interesting to consider the possibility to obtain a polar surface since this can be important for both technical applications and fundamental research. The polar surfaces, however, possess uncompensated net dipole moments perpendicular to the surface, which leads to an increase of the surface free energy making them unstable^13^,^19^. The vast majority of studies of alkali-halide thin films grown on metals^20^,^21^,^22^,^23^,^24^,^25^,^26^,^27^ and semiconductors^8^,^9^,^10^ confirms this fact. However, if some specific requirements are fulfilled, NaCl(111) islands with the uppermost layer corresponding to a polar surface can also be grown, like on Al(111) and Al(001)^2^. In particular, it has been shown that NaCl(111) islands can be grown by adsorption of Na and Cl on such substrates with an excess of Na at the interface^2^. To our knowledge, no other experiments about the formation of (111) alkali halide thin films have been reported so far.

In the present study, the growth of KCl thin films on Si(111)7 × 7 was studied by means of STM under UHV conditions. We demonstrate that the polar (111) surface can be obtained for the KCl films grown at 430 K. We suggest that the formation of the (111) areas occurs as a consequence of the thermally activated dissociation of KCl on Si(111)7 × 7, which produces an excess of potassium on the surface. Moreover, we show that flat and atomically well-defined ultra-thin KCl layers can be epitaxially grown on Si(111)7 × 7.

## Results

**Figure 1:**
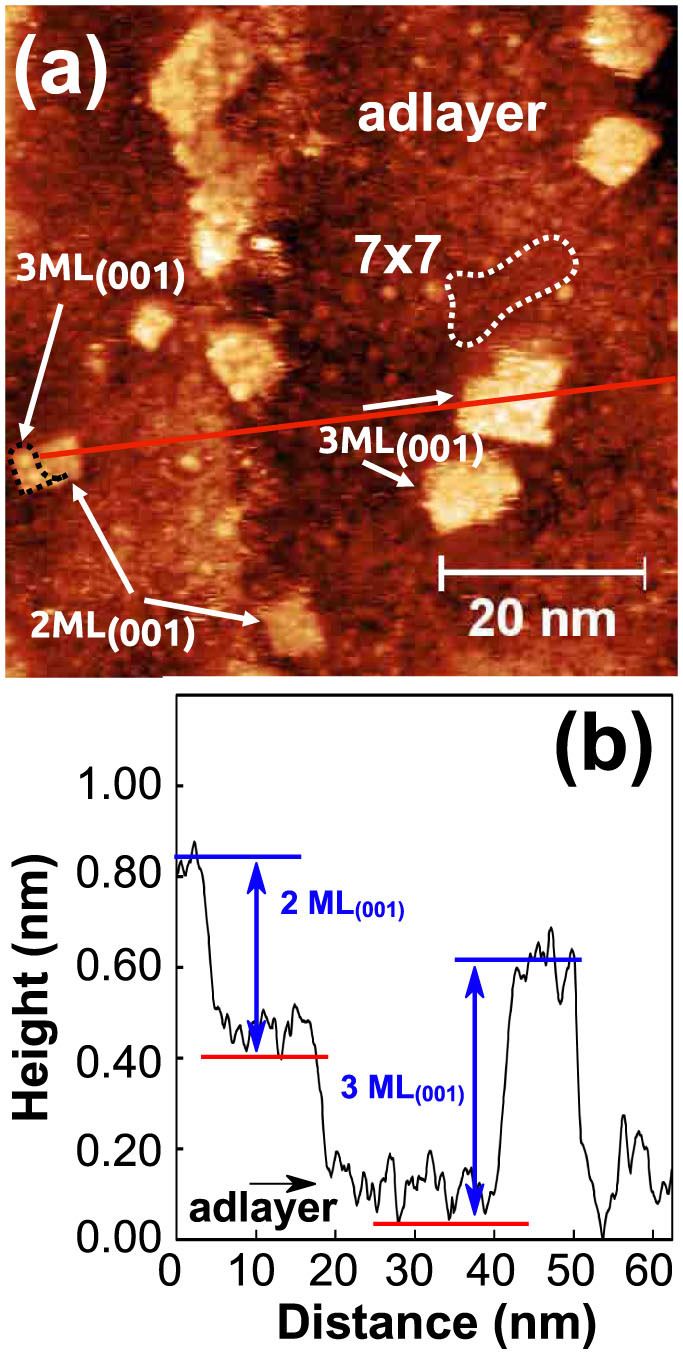
STM topography images of the Si(111)7 × 7 surface in the early stages of KCl growth at room temperature: (a) growth of 2 ML_(001)_ and 3 ML_(001)_ square shaped KCl islands; (b) height profile along the transverse red line in (a). The red lower bar indicates the position of the silicon substrate.

In the early stages of KCl growth at RT, the formation of an almost complete amorphous adlayer with a corrugation of ~1 Å is observed, apart from small regions still showing the Si(111) substrate (see Fig. 1(a), (b) and Supplementary Fig. S1 online). The adlayer is similar to the one observed by Chung et al.^10^ for the NaCl/Si(001) system. Taking their X-Ray photoemission results into account, we expect this adlayer to be composed of KCl molecules, Si-Cl species and K species.

**Figure 2:**
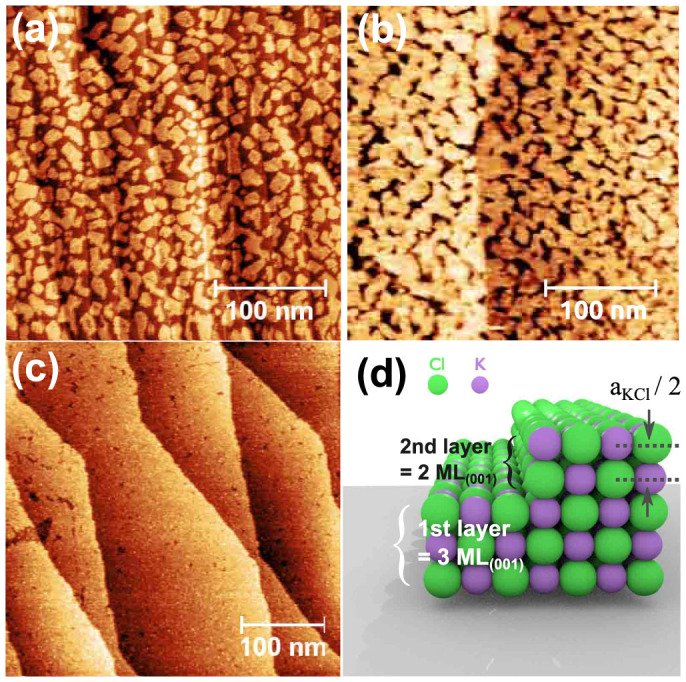
STM topography images of the Si(111)7 × 7 surface after the deposition of KCl at room temperature: (a) growth of 2 ML_(001)_ and 3 ML_(001)_ KCl islands with polygonal shape; (b) and (c) completion of the first and second layers, resulting in a 3 ML_(001)_ (b) and 5 ML_(001)_ ultra-thin KCl film (c), respectively. (d) ball model of the first and second KCl(001) layers.

Further deposition of KCl led to the formation of square shaped islands with different orientations as shown in Fig. 1(a). In the following, we analyze the height of the islands. For this, we define one monolayer of KCl(001) (ML_(001)_) as one atomic layer that contains potassium and chlorine atoms in one plane (see Fig. 2(d)). The height of the ML_(001)_ is the distance between two atomic layers (a_KCl_/2 = 3.15 Å, a_KCl_ = 6.29 Å: unit cell dimension). In Fig. 1(a), two apparent heights of *h*_1_ = (4.5 ± 0.5) Å and *h*_2_ = (6.0 ± 0.5) Å were measured for the islands as exemplified by the profile shown in Fig. 1(b). Note that we measured the height from the silicon substrate as shown by the red lower bar in Fig. 1(b).

The lower apparent height *h*_1_ is well above the one of 3.15 Å expected for 1 ML_(001)_, and clearly below the geometrical 2 ML_(001)_ height of 6.3 Å. The same applies for the height *h*_2_, it lies between the values for 2 ML_(001)_ and 3 ML_(001)_. In STM it is well established that STM imaging of alkali halide ultra-thin films leads to substantially lower apparent height values. The density of states in the band gap is reduced with increasing number of insulating layers and the apparent height of additional layers decreases^1^. We thus attribute the grown square shaped islands to 2 ML_(001)_ and 3 ML_(001)_ KCl islands, as already reported for the growth of NaCl on metal and semiconductor substrates^7^,^8^,^11^,^28^. We note that some 2 ML_(001)_ islands are partially covered by a third atomic layer, suggesting the initial growth of islands of 2 ML_(001)_ thickness prior to the growth of a third ML_(001)_ on top of them.

A subsequent increase of the deposited KCl amount led to a development of the existing 2 ML_(001)_ and 3 ML_(001)_ islands, which exhibit more and more a polygonal shape (see Fig. 2(a)). The edges of the polygonal shaped islands exhibit in most cases angles of 90°, which can deviate due to a coalescence of islands or due to edges that have a high kink density leading to edge orientations other than <001>.

Interestingly, we observed that in many cases the tunneling current from KCl islands was modulated by the 7 × 7 structure lying underneath, i.e. we could still observe the 3-fold symmetry structure originating from the Si(111)7 × 7 reconstructed surface through the KCl islands (see Supplementary Fig. S2(a) online). This strongly indicates that the 7 × 7 reconstruction of the underlying Si(111) surface is unaltered and still intact, similar to the herringbone geometry of the Au(111) surface covered with NaCl islands^11^,^12^.

**Figure 3:**
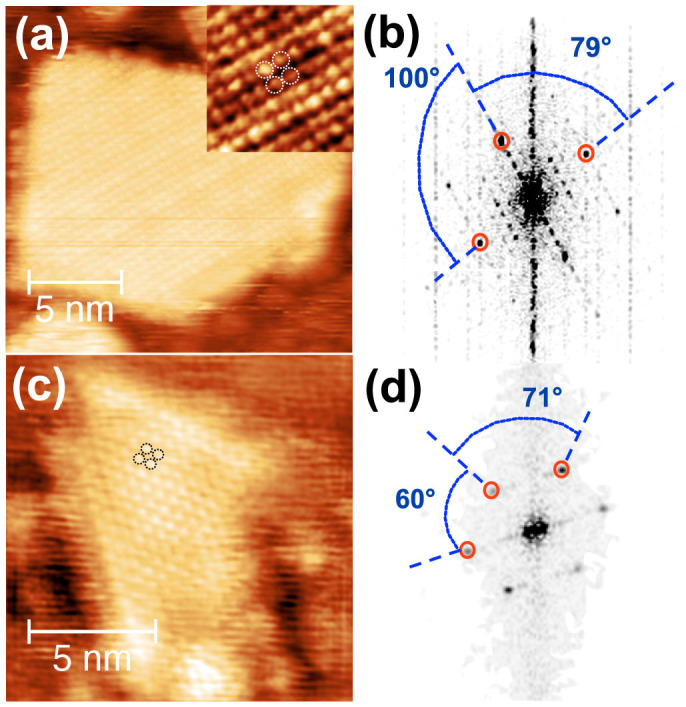
Atomically resolved STM images of (a) polygonal KCl(001) and (c) triangular KCl(111) islands grown at room temperature. The images have been recorded with a sample bias voltage of −2 V and a current of 32 pA. The corresponding 2D FFT images are presented in (b) and (d), respectively.

An atomically resolved STM image of a polygonal 2 ML_(001)_ KCl island is shown in Fig. 3(a). The lattice parameter determined from the 2D FFT of this image (see Fig. 3(b)) equals (4.6 ± 0.3) Å in a good agreement with the sub-cell dimension of either the anions or cations (
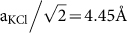
). The angle between the surface vectors was determined to be 90° ± 11°, with the error resulting mainly from the drift of the STM imaging.

**Figure 4:**
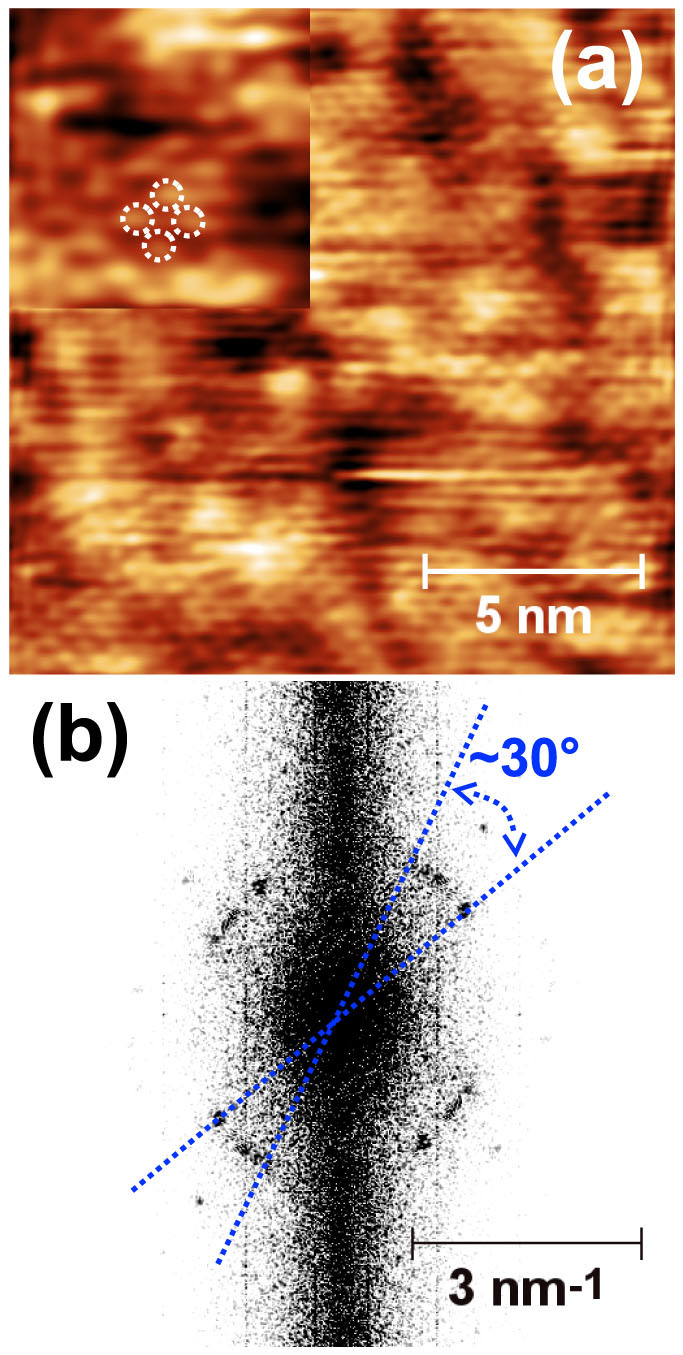
(a) Atomically resolved STM image recorded from the surface of a complete 3 ML_(001)_ KCl film grown at room temperature; (b) corresponding FFT transform (obtained from a larger scale high-resolution image).

Up to the saturation of the Si(111)7 × 7 surface, the growth of the first layer proceeds in a 2 ML_(001)_/3 ML_(001)_ growth mode as already reported for the initial growth of NaCl(001) overlayers on Ge(001)^8^. Prior to the growth of the second layer, an almost complete 3 ML_(001)_ flat layer can be obtained as shown in Fig. 2(b), with an apparent height of ~6 Å (see also Fig. 2(d)). A simple analysis of the high-resolution STM images (as shown in Fig. 4(a)) and the corresponding Fourier transform (see Fig. 4(b)) recorded on the first complete layer confirms that the layer is still in its (001) epitaxy. However, a perfect (001) lattice can only be found in small regions whereas the lattices of the regions differ in their lateral orientations - in other words the film is polycrystalline. A rather high density of defects can be observed, which is a signature that the small initial KCl islands (Fig. 2(a) and (b)) do not all have the same orientation on the Si(111)7 × 7 surface before the films gets closed - when the film closes dislocations and grain boundaries are formed similar to the case of NaCl on Ag(001)^7^. Fig. 2(c) shows an almost closed second layer which displays an apparent height lower than 3 Å above the first layer. This layer can be reasonably assigned to a 2 ML_(001)_ KCl thick layer and the total KCl coverage of the ultra-thin KCl film is thus 5 ML_(001)_ (see Fig. 2(d)). It can be noticed that the density of holes in the second layer is much lower than in the first layer and a flat and homogenous ultra-thin KCl(001) film has thus been formed on the Si(111)7 × 7 substrate.

During the growth of the first and second layers, we found occasionally some small areas corresponding to disruptions of these layers, where triangular shaped islands can be observed. One of such islands and the corresponding 2D FFT are presented in Fig. 3(c) and 3(d). The structure possesses a six-fold symmetry with a lattice constant of (4.6 ± 0.3) Å which agrees well with the lattice parameter of the KCl(111) plane (
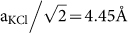
). The triangular islands can thus unambiguously be assigned to KCl(111) islands.

**Figure 5:**
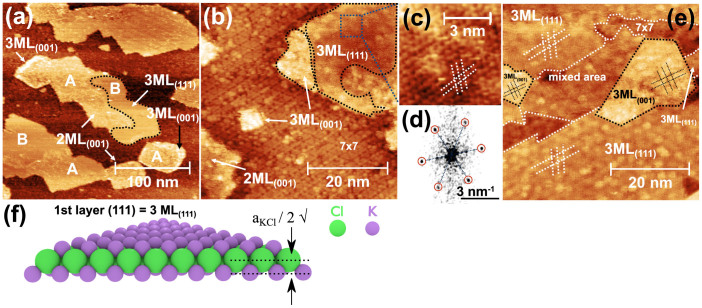
STM topography images of the Si(111)7 × 7 surface after KCl deposition at 400 K (a–b) and 430 K (e): (a) formation of 2 ML_(001)_/3 ML_(001)_ and 3 ML_(111)_ KCl islands; (b) close-up view of 2 ML_(001)_/3 ML_(001)_ and 3 ML_(111)_ islands; (c) higher magnification image of a selected area from the 3 ML_(111)_ area and (d) an FFT obtained from the top of the 3 ML_(111)_ island. (e) After KCl deposition at 430 K: formation of large 3 ML_(111)_ areas, mixed area of (111) and (001) zones and higher 3 ML_(001)_ islands; (f) ball model of a triangular 3 ML KCl(111) island.

To investigate the growth of the KCl thin films at high temperature and to promote the formation of large areas of KCl(111) layers, we performed depositions of KCl at 400 K and 430 K on clean Si(111)7 × 7 surfaces. The amorphous layer described at RT was not observed at elevated growth temperature. Fig. 5(a) presents islands grown at 400 K with clearly two different structures (see areas A and B in Fig. 5(a)). Both structures have the same apparent height of (4.0 ± 0.5) Å within the error bars. The first structure A can clearly be associated with that of small square shaped islands and thus assigned to 2 ML_(001)_ KCl islands. We also observe the presence of small square shaped islands with an apparent height of (6.0 ± 0.5) Å assigned to 3 ML_(001)_ islands and 3 ML_(001)_ patches at the edges of islands (see Fig. 5(a) and (b)). The analysis of the atomically resolved STM images and the corresponding 2D FFT (see Fig. 5(b) to (d)) unambiguously reveal that the second structure B possesses a six-fold symmetry, yielding 60° and 120° islands corners and a lattice constant close to 4.5 Å, which corresponds to the lattice of either the Cl or Na ions in the (111) plane. Thus, we assign the second structure B to KCl(111). Note that we define a monolayer (1 ML_(111)_) as an atomic layer that contains either only potassium or chlorine atoms (Fig. 5(f)). The height of the ML_(111)_ is then the distance between two atomic layers (
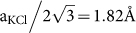
), and multiple layers will be an integer multiple of the latter height. The apparent height of the B islands is well above the physical thickness of 2 ML_(111)_ KCl layers (3.64 Å) and lower than the geometrical 3 ML_(111)_ height of 5.46 Å, suggesting that the KCl(111) areas consist of 3 ML_(111)_. We note that formation of similar alkali halide structures have been observed before, consisting of 3 ML_NaCl(111)_ islands grown on Al(111) and Al(001)^2^. Interestingly, the 7 × 7 structure can be distinguished in STM images underneath the KCl(111) islands, which strongly indicates that the growth of the (111) KCl structure does not alter the 7 × 7 reconstruction (see Supplementary Fig. S2(b) online). For all KCl(111) islands, the surface directions of the islands are the same as for the Si(111) surface, e.g. 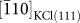 and 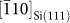 are parallel.

A higher deposition temperature (430 K) favors the growth of very large KCl(111) areas on the Si(111)7 × 7 surface as shown by high resolution STM images (see Fig. 5(e) and Supplementary Fig. S3 and S4 online) and supported by 2D FFT of such areas. In agreement with the above discussion, we assign these KCl(111) areas to 3 ML_(111)_ (see Fig. 5(f)). Apart from the very large (111) areas we observe, however, also small areas where the film has a higher thickness, with an apparent height difference of ~2 Å above the (111) layer (see Fig. 5(e) and Supplementary Fig. S3 and S4 online). Such regions are either square shaped or have a polygonal shape. Even though the atomic structure of these islands appears less organized than in the RT case, 2D FFT confirms that these higher islands correspond to KCl(001). In agreement with our observations at RT and 400 K, we assigned these higher islands to 3 ML_(001)_ islands. It can be further noted that small atomically poorly organized areas have been also formed (denoted mixed area in Fig. 5(e)), which could correspond to mixed (111) and (001) zones.

## Discussion

We will focus in the following on the mechanisms which could explain the growth of (111) areas at high temperature. It has been reported that the formation of NaCl(111) islands is possible on Al(111) and Al(001) by conversion of NaCl(001) to NaCl(111) upon Na post-deposition onto a stoichiometric NaCl(001) film with the sample kept at RT^2^. The obtained islands possess Na-Cl-Na triple layer structure. In our experiments, the apparent height of the (111) layers is consistent with the thickness of a 3 ML_(111)_ layer. We thus assume that such layers have a K-Cl-K structure, in analogy with the model reported by Hebenstreit et al.^2^. The formation of KCl(111) areas in our experiments would thus result from an excess of potassium on the surface during the growth. A possible explanation for the formation of the K rich islands could be that the beam of KCl was non-stoichiometric. The non-stoichiometry could be due to the formation of an excess of K^+^ and 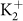 ions as well as non-stoichiometric K_2_Cl^+^ and 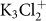 cations in the molecular beam as stated by Butman *et al.*^29^. However, in the latter work, it has been shown that the nature of the evaporated species drastically depends on the evaporation source: in the case of a Knudsen cell, K_2_Cl^+^ but not K^+^ and 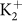 ions are the dominant emitted species^30^. Note that, a non-stoichiometric KCl beam does not explain the increased fraction of the (111) areas at 400 K and 430 K. Therefore, we stress that the KCl beam is not the source of the potassium excess on our surfaces.

Earlier photoemission studies on KCl/Si(001)^9^ and NaCl/Si(001)^10^ at 330 K have shown that KCl and NaCl partially dissociate upon adsorption on the silicon surface to form Si-Cl bonds and adsorbed K and Na. We therefore assume that a similar process occurs during the KCl adsorption on the Si(111)7 × 7 substrate. Desorption of Cl in form of SiCl_2_ and SiCl_4_ species from Si(111)7 × 7, inducing etching of the substrate, is reported at slightly higher temperatures (starting at ~330 K) than RT^31^ and with respect to K, significant desorption via first and second order processes, occurs at temperatures higher than those used in our experiments (up to 430 K)^32^,^33^. Thus, we suggest that adsorption of KCl on the Si(111)7 × 7 surface leads to an excess of K on the surface via a dissociation of KCl, allowing the formation of 3 ML_(111)_ K-Cl-K areas. The percentage of surface areas between (111) and (001) zones, measured in STM images is (5 ± 2)% at RT, (40 ± 10)% at 400 K and (80 ± 10)% at 430 K. The increased fraction of the (111) areas with the increase of the growth temperature, at constant flux, suggests that this process of KCl dissociative adsorption is thermally activated, leading to an increase of the K excess on the surface with increasing temperature. We can therefore assume that at the high temperature of 430 K, the majority of islands grows in a (111) epitaxy, whereas only a small fraction of (001) islands grows at the same time on the Si substrate. We suggest that, apart from the thermally activated dissociative adsorption of KCl, the silicon substrate is needed, which facilitates the dissociation of KCl and produces an excess of K on the surface.

## Conclusions

In conclusion, we have conducted an in-situ characterization of the atomic structure and morphology of KCl films grown on Si(111)7 × 7 by means of STM. Our work shows that the stacking, termination and consequently the polarity of such films can be tuned by adjusting the growth temperature - an approach that can most likely be extrapolated also to other alkali halide thin films on Si(111)7 × 7. Such KCl films with controllable polarity are suitable for the growth of organic molecules which are electronically decoupled from the conductive Si(111) substrate. Furthermore, these ultra-thin layers can be successfully employed for the growth and the subsequent removal of metal nanoparticles from the Si substrate by dissolution of the KCl layer in liquid as it will be shown in a forthcoming paper.

## Methods

Ultra-thin KCl films have been grown under UHV (base pressure 3 · 10^−10^ mbar) on n-doped Si(111) substrates misoriented by 1° towards the 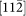 direction. The 7 × 7 reconstructed (111) surface was obtained using a standard heating procedure described elsewhere^34^. KCl powder was evaporated from a Knudsen cell with a molybdenum crucible using a commercial electron beam Omicron EFM 3T evaporator. KCl depositions were performed with the sample kept at room (RT) and high temperatures (400 K–430 K) with a constant deposition rate of ~2 ML_(001)_/min.

In the case of KCl(001), one monolayer of KCl(001) (ML_(001)_) is defined to be one atomic layer that contains potassium and chlorine atoms. In this case the monolayer is stoichiometric, and the height of the ML_(001)_ is the distance between two atomic layers (a_KCl_/2, a_KCl_ = 6.29 Å: unit cell dimension) with multiple layers being an integer multiple of the latter height. In the case of KCl(111), we define for simplicity reasons a monolayer (ML_(111)_) as an atomic layer that contains either only potassium or chlorine atoms. Note that in this case, a monolayer is certainly not stoichiometric. The height of the ML_(111)_ is then the distance between two atomic layers (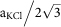), and multiple layers will be an integer multiple of the latter height.

For the depositions at high temperatures, resistive DC heating was applied to the sample. The temperature readings were calibrated using an IR pyrometer for the spot where the STM measurements were performed. The accuracy of the temperature measurement is ±20 K. The samples were examined in-situ by an Omicron™ VT-STM operating in the constant current mode using chemically etched tungsten tips. The optimal sample bias was in the range of −1.7 V to −2.1 V, whereas the setpoint was below ~300 pA. We would like to mention that the imaging was only possible with negative sample bias voltages. Atomically resolved STM images were analyzed in terms of two-dimensional (2D) fast Fourier transforms (FFT) using the plane-fitted STM data processed in Gwyddion^35^.

## Author Contributions

L.M. and M.H. are at the origin of the scientific project. I.B. and L.M. conceived the experiments, performed and analyzed the measurements. I.B., C.B. and L.M. provided the interpretation of the results. I.B., C.B., M.H. and L.M. contributed to writing of the article.

## Supplementary Material

Supplementary Information KCl ultra-thin films with polar and non-polar surfaces grown on Si(111) 7x7
