# Supplementary material for: KCl ultra-thin films with polar and non-polar surfaces grown on Si(111)7 × 7
Source: Sci Rep. 2015 Feb 4;5:8223. doi: 10.1038/srep08223 (PMC4316162; doi:10.1038/srep08223)
Supplement: Supplementary Information — KCl ultra-thin films with polar and non-polar surfaces grown on Si(111) 7x7 [file srep08223-s1.pdf]

KCl ultra-thin films with polar and non-polar surfaces grown on Si(111)7×7

Igor Beinik[1], Clemens Barth[1], Margrit Hanbücken[1] and Laurence Masson[1,*]

[1]*Aix Marseille Université, CNRS, CINaM UMR 7325, F-13288, Marseille, France*

In this Supplementary Information, we present additional scanning tunneling microscopy (STM) images describing the adsorption of KCl on Si(111)7×7 at room temperature (RT), 400 K and 430 K.

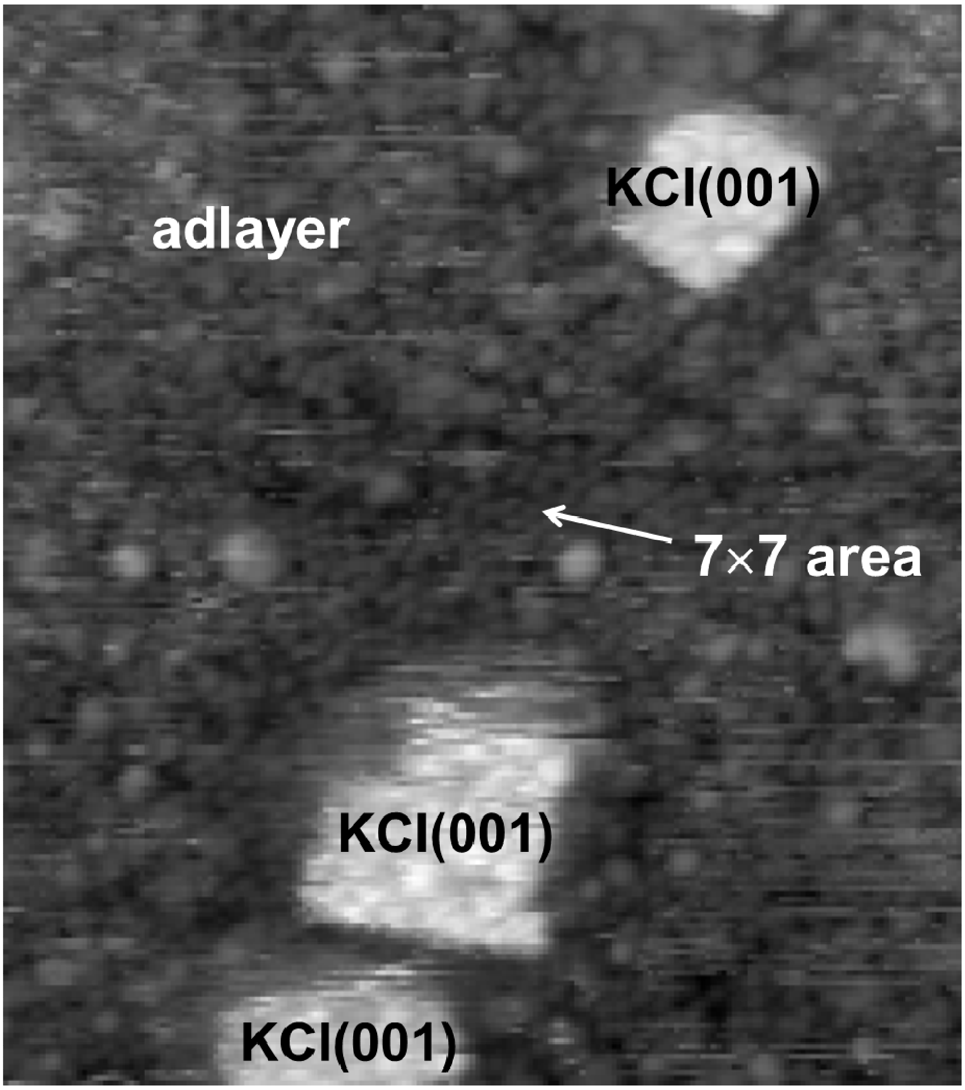

Figure S1. Zoom-in of the STM image displayed in Fig. 1(a) (35×40 nm^2) showing the almost complete adlayer formed in the early stages of KCl growth at RT, prior to the growth of KCl(001) islands, and an uncovered area of the Si(111)7×7 substrate.

The STM image shown in Fig. S2(a) has been acquired after the one presented in Fig. 3(a), recorded with the same tunneling conditions. Variations between the two STM images may be ascribed to modifications of the STM tip apex, causing changes in the tip electronic states and therefore in the STM image itself.

Figure S2(b) corresponds to an STM image of one of the area B of KCl islands in Fig. 5(a) corresponding to 3 $ML_{(111)}$ area.

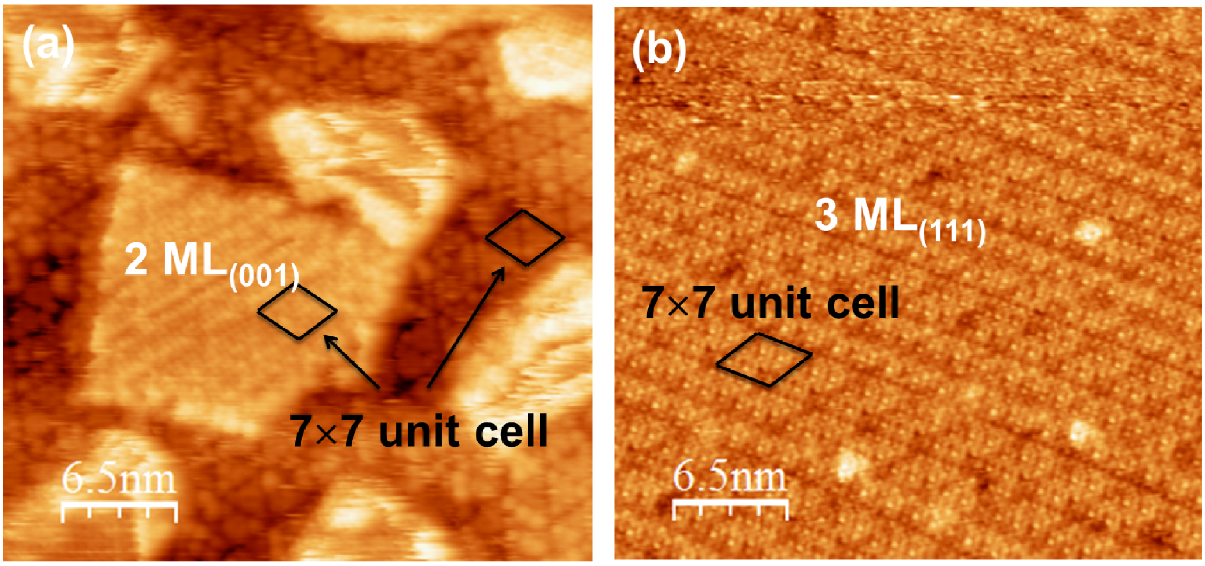

Figure S2. STM images of KCl islands after KCl adsorption on Si(111)7×7 at (a) RT (sample bias voltage= -2 V , current = 32 pA) and (b) 400 K (sample bias voltage= -2.1 V , current = 39 pA). In both images, the 7×7 structure can been distinguished underneath the KCl islands. Thermal drift was not corrected.

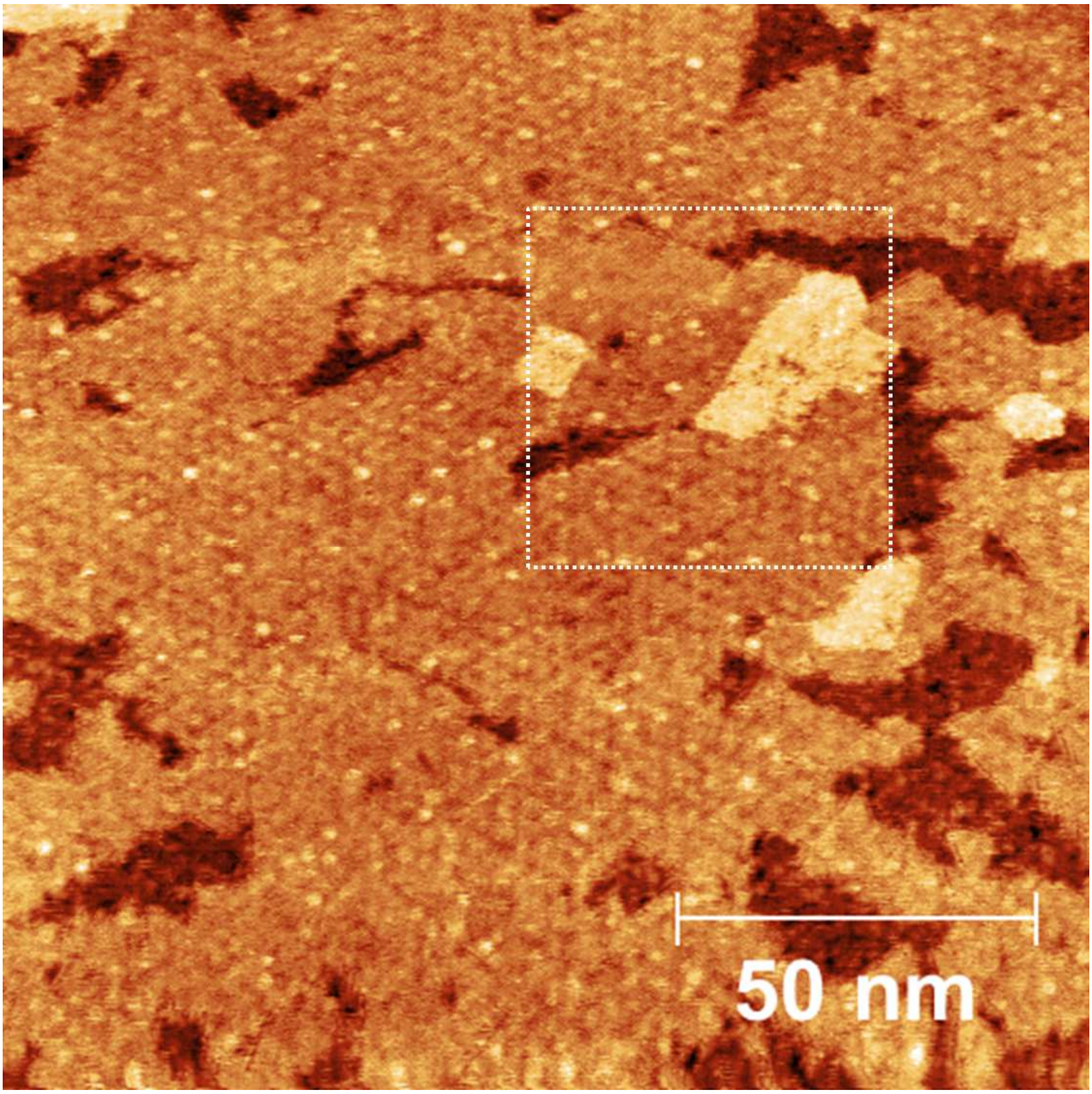

Figure S3. KCl adsorption on Si(111)7×7 at 430 K. STM image showing the growth of an almost complete first layer on Si(111)7×7. The square (dashed) indicates the selected area shown in Fig. S4.

Figure S4 corresponds to an enlarged scale of the STM image displayed in Fig. 5(e) to show details of the atomic structure of the first layer covering the silicon substrate, essentially composed of large (111) areas.

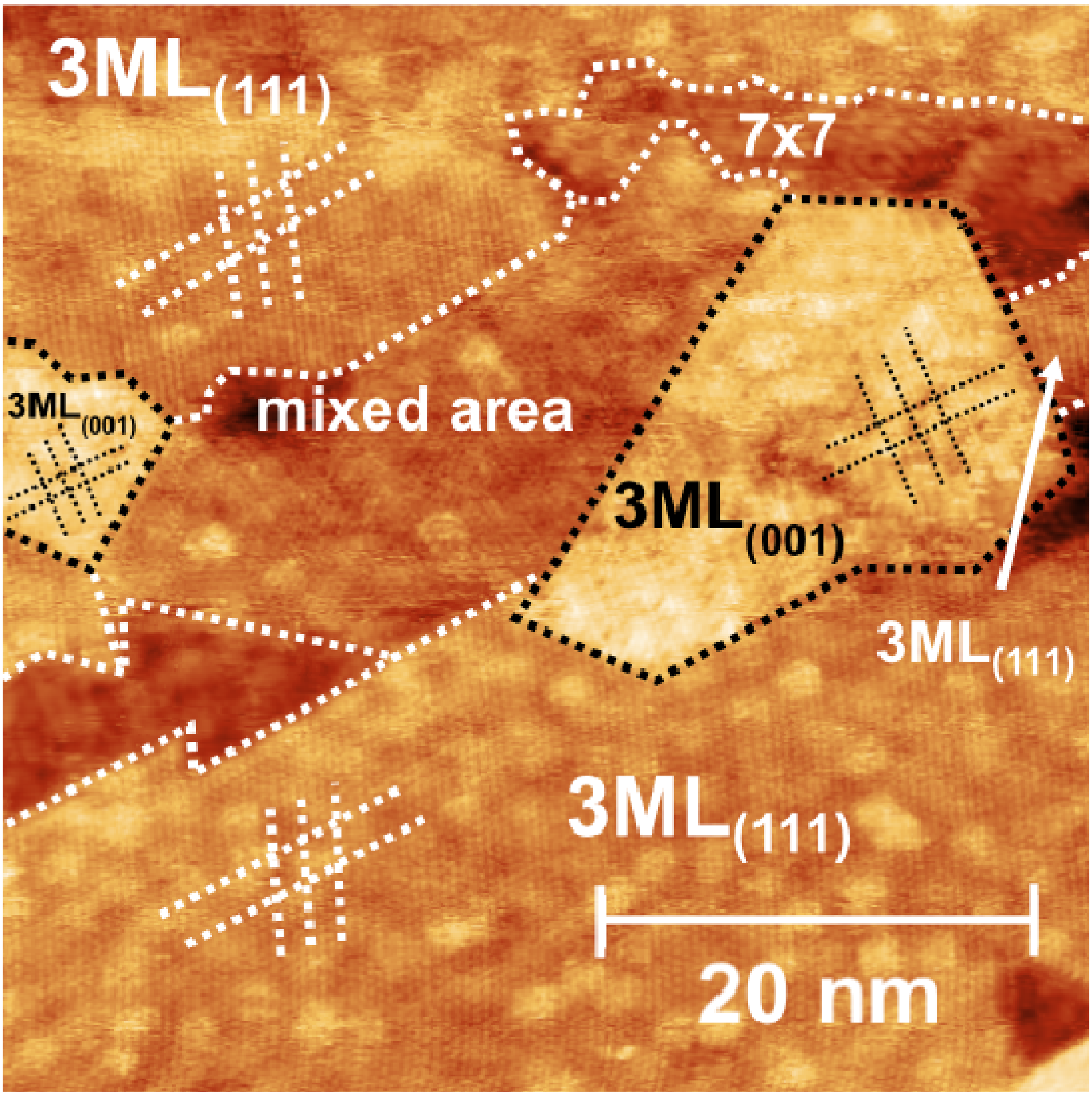

Figure S4. KCl adsorption on Si(111)7×7 at 430 K. High resolution STM image of a selected area in Fig. S3 showing large 3 $ML_{(111)}$ areas, mixed area of (111) and (001) zones and higher 3 $ML_{(001)}$ islands.
